# Association of newer definitions of bronchopulmonary dysplasia with pulmonary hypertension and long-term outcomes

**DOI:** 10.3389/fped.2023.1108925

**Published:** 2023-02-15

**Authors:** Jae Kyoon Hwang, Seung Han Shin, Ee-Kyung Kim, Seh Hyun Kim, Han-Suk Kim

**Affiliations:** ^1^ Department of Pediatrics, Hanyang University Guri Hospital, Gyeonggi-do, Republic of Korea; ^2^ Department of Pediatrics, Seoul National University College of Medicine, Seoul, Republic of Korea

**Keywords:** bronchopulmonary dysplasia, developmental delay, prognosis, pulmonary hypertension, readmission

## Abstract

**Background:**

The definition of bronchopulmonary dysplasia (BPD) has been evolved recently from definition by the National Institute of Child Health and Human Development in 2001 (NICHD 2001) to the definition reported in 2018 (NICHD 2018) and that proposed by Jensen et al. in 2019 (NICHD 2019). The definition was developed based on the evolution of non-invasive respiratory support and to achieve better prediction of later outcomes. Our objective was to evaluate the association between different definitions of BPD and occurrence of pulmonary hypertension (PHN) and long term outcomes.

**Methods:**

This retrospective study enrolled preterm infants born at < 32 weeks of gestation between 2014 and 2018. The association between re-hospitalization owing to a respiratory illness until a corrected age (CA) of 24 months, neurodevelopmental impairment (NDI) at a CA of 18–24 months, and PHN at a postmenstrual age (PMA) of 36 weeks was evaluated, with the severity of BPD defined based on these three definitions.

**Results:**

Among 354 infants, the gestational age and birth weight were the lowest in severe BPD based on the NICHD 2019 definition. In total, 14.1% of the study population experienced NDI and 19.0% were re-hospitalized owing to a respiratory illness. At a PMA of 36 weeks, PHN was identified in 9.2% of infants with any BPD. Multiple logistic regression analysis showed that the adjusted odds ratio (OR) for re-hospitalization was the highest for Grade 3 BPD of the NICHD 2019 criteria (5.72, 95% confidence interval [CI]: 1.37–23.92), while the adjusted OR of Grade 3 BPD was 4.96 (95% CI: 1.73–14.23) in the NICHD 2018 definition. Moreover, no association of the severity of BPD was found in the NICHD 2001 definition. The adjusted ORs for NDI (12.09, 95% CI: 2.52–58.05) and PHN (40.37, 95% CI: 5.15–316.34) were also the highest for Grade 3 of the NICHD 2019 criteria.

**Conclusion:**

Based on recently suggested criteria by the NICHD in 2019, BPD severity is associated with long-term outcomes and PHN at a PMA of 36 weeks in preterm infants.

## Introduction

Bronchopulmonary dysplasia (BPD) is a chronic lung disease that occurs in preterm infants and is one of the most important morbidities that determine later health outcomes in this population. Since it was first described in 1967 (1), the definition and characteristics of the disease have evolved over the past 50 years as more immature infants can now survive. To date, the definition of BPD proposed in 2001 in a consensus conference of the National Institute of Child Health and Human Development (NICHD) is the most commonly used, and this categorized BPD severity based on the mode of respiratory support and the amount of supplemental oxygen required (2). Subsequently, a physiological definition of BPD has been suggested as the degree of respiratory support determined by each attending physician, rather than according to the basis of functional assessment (3). The diagnosis and severity of BPD have been associated with long-term adverse effects on respiratory, neurodevelopmental, and growth outcomes and contributes to early mortality as well (4, 5).

However, most commonly used definitions have some limitations (6). One of the most important issues is the fact that it does not incorporate recently introduced non-invasive respiratory support, such as high-flow nasal cannula, as those have been important strategies in the care of preterm infants (7, 8). In this context, revised refinements of the definition of BPD were suggested in the NICHD workshop in 2018, which considered non-invasive positive pressure ventilation (NIPPV) and heated humidified high flow nasal cannula as important modalities (9). More recently, Jensen et al. explored various criteria for diagnosing BPD (NICHD 2019) not only in view of current practices, but also in terms of the ability to accurately predict later health outcomes (10).

Moreover, etiology and pathophysiology of the disease were not considered in the BPD criteria in 2001 (6). Especially, pulmonary vascular disease has been recently recognized as an important pathophysiology of BPD in neonates born with immature lungs, and the association of BPD with this condition has been demonstrated as there is a relatively high prevalence of pulmonary hypertension (PHN) in patients with severe BPD (11, 12). Although it is difficult to include PHN in the current definition of BPD, a better-categorized definition of BPD may identify the association of PHN more clearly in severe forms of the disease.

In this study, the three criteria for diagnosing BPD in preterm infants, including the NICHD 2001, NICHD 2018, and NICHD 2019, were used to predict respiratory and neurodevelopmental outcomes at a corrected age (CA) of 18–24 months. Furthermore, the association between BPD severity and PHN at a postmenstrual age (PMA) of 36 weeks was explored using each criterion.

## Methods

### Population and data source

This retrospective study enrolled preterm infants who were born at < 32 weeks' gestation at our institution, between January 2014 and December 2018. Infants with congenital anomalies or congenital infections and infants who died before being discharged were excluded from the study population. Those who were lost to follow-up at a CA of 18–24 months were also excluded. Data on perinatal characteristics, clinical courses, PHN, and mode of respiratory support at a PMA of 36 weeks were collected. PHN was diagnosed at a PMA of 36 weeks using echocardiography based on the following findings: right-to-left or bidirectional shunt *via* patent ductus arteriosus or patent foramen ovale, velocity of tricuspid regurgitation ≥ 3 m/s, and left-deviated or flat configuration of the interventricular septum. Re-hospitalization due to a respiratory illness until a CA of 24 months and neurodevelopmental outcomes at a CA of 18–24 months were evaluated. Follow-up results were collected from the data achieved during regular outpatient follow-up schedule until 48 months of age, according to the institution's protocol. Hwang JK and Shin SH collected and reviewed the data from the electric medical records, and retrospectively categorized severity of BPD based on the three criteria. This study was approved by the Institutional Review Board of our institution (2109-008-1250). Obtaining informed consent was waived by the Institutional Review Board, and all methods were performed in accordance with the guidelines of the Human Research Protection Program.

### Grading the severity of BPD based on the NICHD 2001, NICHD 2018, and NICHD 2019 criteria

First, BPD was diagnosed and graded as a mild, moderate, or severe disease according to the definition established by the NICHD in 2001 (NICHD 2001) (2). Second, the recently proposed skeletal definition of BPD by the NICHD in 2018 was used (NICHD 2018) (9). In 2019, Jensen et al. compared a number of diagnostic criteria for BPD, in terms of various types of non-invasive respiratory support (NICHD 2019) (10). Among the criteria, those that most accurately predicted later health outcomes were adopted in this study. These were used to categorize BPD that required respiratory support with low-flow (≤2 L/min) nasal cannula at PMA of 36 weeks (Grade 1); BPD that required high flow (>2 L/min) nasal cannula, nasal continuous positive airway pressure, or NIPPV at a PMA of 36 weeks (Grade 2); and BPD that required invasive positive pressure ventilation at a PMA of 36 weeks (Grade 3) (NICHD 2019). Supplementary Table S1 compares the three definitions at a glance (Supplementary Table S1).

### Outcomes

Pulmonary hypertension was diagnosed with echocardiography at a PMA of 36 weeks, based on the presence of at least one of the following criteria: (1) velocity of tricuspid valve regurgitation ≥ 3 m/s in the absence of pulmonary stenosis or (2) flat or left-deviated interventricular septal configuration and right ventricular hypertrophy with chamber dilation (13). Respiratory morbidity was defined as re-hospitalization due to a respiratory illness until a CA of 24 months in the study institution and other hospitals; this information was obtained as part of the routine follow-up protocol, based on information provided by the caregiver. The Bayley Scales of Infant and Toddler Development 3rd Edition (Bayley-III) results at a CA of 18–24 months were reviewed; scores < 85 (-1 SD) points in both cognitive and language domains or a motor score < 85 points were defined as developmental delay (14). The test was conducted by one nurse practitioner who has been appropriately trained and specialized for Bayley-III. Combined neurodevelopmental impairment (NDI) was defined when there was any of the following: blindness, hearing impairment that required the use of hearing aids, cerebral palsy, and developmental delay in Bayley-III. Sepsis was defined if pathogen was demonstrated in blood culture and required systemic antibiotic treatment for more than 5 days. Periventricular leukomalacia was defined when cystic or non-cystic findings of white matter injury was found in imaging tests, such as ultrasound and magnetic resonance imaging (MRI) (13). Intraventricular hemorrhage (IVH) was defined according to Papile's classification (15).

### Statistical analyses

Fisher's exact test was used for categorical variables, and one-way analysis of variance was conducted to compare continuous variables according to the severity of BPD with the post-hoc test of Bonferroni correction using the NICHD 2019 criteria. Gestational age, birth weight *z*-score, sex, sepsis, IVH (grade ≥ 3), and periventricular leukomalacia were adjusted in the multivariate logistic regression analysis for later respiratory and neurodevelopmental outcomes as well as PHN at a PMA of 36 weeks based on the three different definitions of BPD. Values are expressed as numbers (%) or medians (interquartile ranges), and a *p*-value < 0.05 indicated statistical significance. The STATA 12.0 software for Windows (Stata Corp., College Station, TX, United States) was used to analyze all data.

## Results

During the study period, 479 infants who had <32 weeks' gestation were born at our facility. Infants with congenital anomalies, congenital infection, and died before discharged were excluded. Infant who died after being discharged and was lost to follow-up at a CA of 18–24 months were also excluded; the remaining 354 infants were included in the final analysis (Supplementary Figure S1).

Demographic findings of the study population are summarized according to the severity of BPD based on the NICHD 2019 (Table 1), NICHD 2001, and NICHD 2018 criteria (Supplementary Tables S2,S3). Based on the NICHD 2019 definition, gestational age (26.9 [25.3–29] weeks) and birth weight (730 [620–980] g) were the lowest among infants with severe BPD, but the incidence of small for gestational age was the highest among infants with severe BPD (20.8%). While the prevalence of multiple births was the highest among infants without BPD (74.3%), histologic chorioamnionitis and oligohydramnios were most common in the severe BPD group (62.3% and 35.8%, respectively). The prevalence of respiratory distress syndrome, patent ductus arteriosus requiring treatment, high-grade IVH, retinopathy of prematurity operation, and PHN at a PMA of 36 weeks were the highest in infants with severe BPD based on the NICHD 2019 (Table 2), NICHD 2001, and NICHD 2018 criteria (Supplementary Tables S4,S5).

**Table 1:** Demographics of the study population according to the severity of bronchopulmonary dysplasia (NICHD 2019).

	No BPD (*n* = 269)	Grade 1 (*n* = 37)	Grade 2 (*n* = 39)	Grade 3 (*n* = 9)	*p*-value
GA (week)	30.3 (28.9–31.1)	28.1 (26.7–29.5)	26.4 (24.4–28.7)	27.6 (25.3–30.2)	<0.001
Birth weight (g)	1,290 (1075–1500)	980 (720–1220)	710 (600–990)	800 (645–1095)	<0.001
Birth weight *z*-score	−0.1 (−0.6–0.4)	−0.3 (−1.1–0.4)	−0.3 (−1.1–0.3)	0.0 (−1.6–0.5)	0.053
SGA	19 (7.1)	8 (21.6)	7 (17.9)	2 (22.2)	0.007
Male	136 (50.6)	22 (59.5)	18 (46.2)	3 (33.3)	0.470
C/S	155 (57.6)	21 (56.8)	26 (66.7)	4 (44.4)	0.593
Multiple birth	190 (70.6)	14 (37.8)	19 (48.7)	4 (44.4)	<0.001
hCAM	103 (38.6)	14 (38.9)	28 (71.8)	5 (55.6)	0.001
PROM	109 (40.8)	14 (37.8)	20 (52.6)	4 (44.4)	0.535
Oligohydramnios	48 (17.8)	8 (21.6)	14 (35.9)	5 (55.6)	0.004
Antenatal steroid	227 (84.4)	34 (91.9)	39 (100.0)	7 (77.8)	0.033

Values are expressed as numbers (%) or medians (interquartile ranges).

BPD, bronchopulmonary dysplasia; NICHD, National Institute of Child Health and Human Development; GA, gestational age; SGA, small for gestational age; C/S, Cesarean section; hCAM, histologic chorioamnionitis; PROM, premature rupture of membranes; NDI; neurodevelopmental impairment.

**Table 2:** Clinical courses according to the severity of BPD (NICHD 2019).

	No BPD (*n* = 269)	Grade 1 (*n* = 37)	Grade 2 (*n* = 39)	Grade 3 (*n* = 9)	*p*-value
RDS	118 (43.9)	27 (73)	33 (84.6)	7 (77.8)	<0.001
PDA treated	56 (27.9)	21 (58.3)	24 (64.9)	7 (77.8)	<0.001
IVH (grade ≥3)	4 (1.5)	1 (2.7)	6 (15.4)	0 (0)	<0.001
NEC	7 (2.6)	2 (5.4)	4 (10.3)	1 (11.1)	0.079
ROP operation	5 (1.9)	3 (8.1)	19 (48.7)	5 (55.6)	<0.001
PHN at a PMA 36 weeks	2 (1.4)	3 (8.1)	6 (15.4)	4 (44.4)	<0.001

Values are expressed as numbers (%) or medians (interquartile ranges).

BPD, bronchopulmonary dysplasia; NICHD, National Institute of Child Health and Human Development; RDS, respiratory distress syndrome; PDA, patent ductus arteriosus; IVH, intraventricular hemorrhage; NEC, necrotizing enterocolitis; ROP, retinopathy of prematurity; PHN, pulmonary hypertension; PMA, postmenstrual age.

In total, 50 (14.1%) infants experienced combined NDI at a CA of 18–24 months and 67 (19.0%) were re-hospitalized owing to a respiratory illness Table 3). Re-hospitalization due to a respiratory illness was higher in Grade 2 and Grade 3 BPD (38.5% and 55.6%, respectively) groups than in the no BPD group (14.1%). Scores of cognitive, language, and motor domains in Bayley-III were the lowest in Grade 3 BPD group compared with the other groups (85 [75–95], 83 [74–91], and 88 [70–94] points, respectively). Delays in both cognitive and language domains, as well as in the motor domain were most common in Grade 3 BPD group (42.9% and 42.9%, respectively). Cerebral palsy occurred most frequently in the severe BPD group (11.1%), but there were no cases of blindness or hearing impairment that required the use of hearing aids.

**Table 3:** Neurodevelopmental and respiratory outcomes according to the severity of BPD (NICHD 2019).

	No BPD (*n* = 269)	Grade 1 (*n* = 37)	Grade 2 (*n* = 39)	Grade 3 (*n* = 9)	*p*-value
Re-hospitalization	35 (13)	12 (32.4)	15 (38.5)§	5 (55.6)§	<0.001
Bayley-III					
Cognitive	100 (90–110)	95 (90–100)	85 (80–95)§	85 (75–95)	<0.001
Language	97 (89–109)	91 (83–100)	83 (71–94)§	83 (74–91)	<0.001
Motor	100 (91–107)	97 (88–98.5)	91 (79–94)§	88 (70–94)§	<0.001
Cognitive and Language <85	13 (6.7)	4 (14.3)	8 (22.9)	3 (42.9)§	0.001
Motor <85	14 (7.3)	5 (17.9)	12 (34.3)§	3 (42.9)	<0.001
CP	10 (3.7)	0 (0)	4 (10.3)	1 (11.1)	0.039
Blindness	–	–	–	–	
Hearing aids	–	–	–	–	
Combined NDI	24 (8.9)	5 (13.5)	16 (41)§*	5 (55.6)§*	<0.001

Values are expressed as numbers (%) or medians (interquartile ranges). BPD, bronchopulmonary dysplasia; NICHD, National Institute of Child Health and Human Development; Bayley-III, Bayley Scales of Infant and Toddler Development 3rd Edition; CP, cerebral palsy; NDI, neurodevelopmental impairment.

^§^ Indicates *p* < 0.013 compared with the no BPD group in the post-hoc analysis with Bonferroni correction.

*Indicates *p* < 0.013 compared with the mild BPD group in the post-hoc analysis with Bonferroni correction.

**Figure 1:**
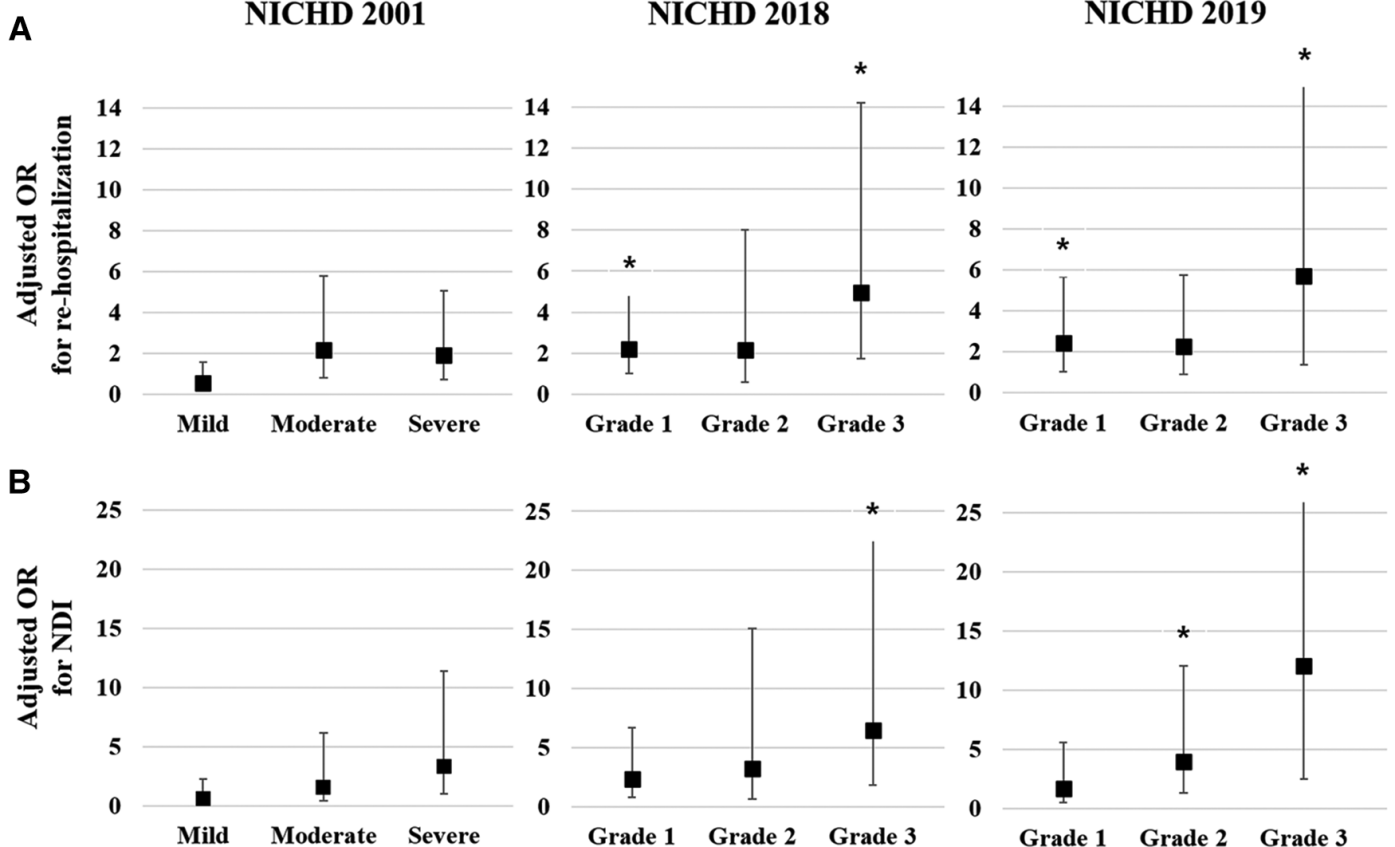
Multivariate analysis for respiratory and neurodevelopmental outcomes at a CA of 18–24 months based on each of the criteria for BPD. Gestational age, birthweight *z*-score, and sex were adjusted in the multivariate analysis. Adjusted OR of the severity of BPD based on the three criteria for re-hospitalization owing to a respiratory illness until a CA of 18–24 months (**A**). Adjusted OR of the severity of BPD based on the three criteria for NDI at a CA of 18–24 months (**B**). Asterisk (*) shows significantly adjusted OR (*p* < 0.05). CA, corrected age; BPD, bronchopulmonary dysplasia; OR, odds ratio; NDI, neurodevelopmental impairment; NICHD, National Institute of Child Health and Human Development.

**Figure 2:**
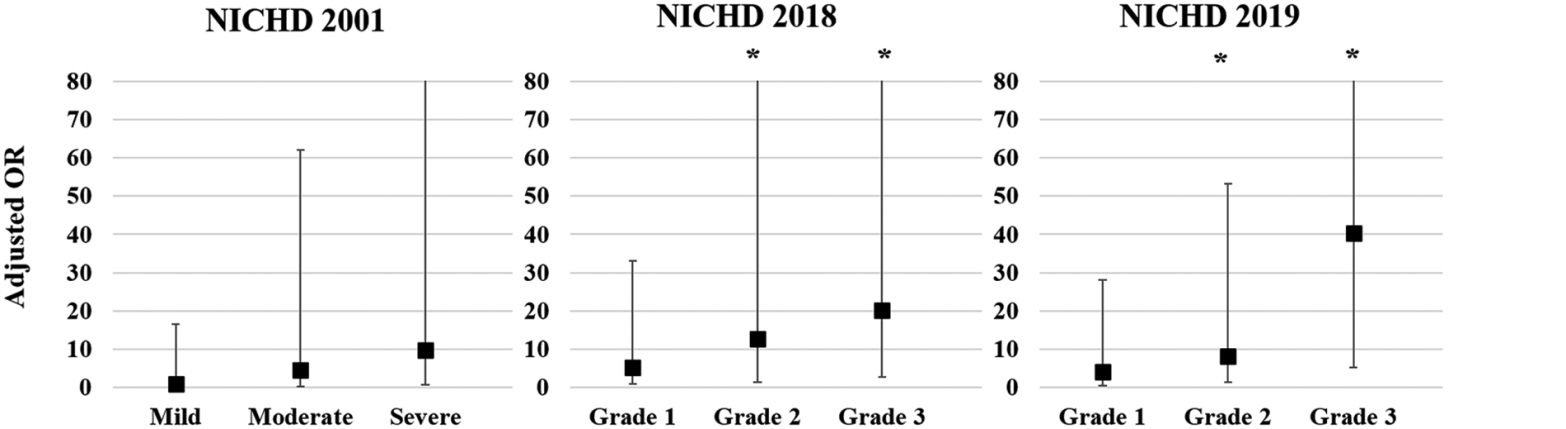
Adjusted OR for PHN at a PMA of 36 weeks based on each of the criteria for BPD. Gestational age, birthweight *z*-score, and sex were adjusted in the multivariate analysis. Asterisk (*) shows significant adjusted OR (*p* < 0.05). OR, odds ratio; PHN, pulmonary hypertension; PMA, postmenstrual age; BPD, bronchopulmonary dysplasia; NICHD, National Institute of Child Health and Human Development.

Multivariate logistic regression analysis was conducted to calculate the adjusted odds ratio (OR) for re-hospitalization and combined NDI based on the three different criteria for BPD Figure 1). The adjusted OR for re-hospitalization was the highest for Grade 3 in the NICHD 2018 (4.96, 95% confidence interval [CI]: 1.73–14.23) and Grade 3 in the NICHD 2019 (5.72, 95% CI: 1.37–23.92), while no association was found in the NICHD 2001 definition (Figure 1A). Adjusted OR for combined NDI was also the highest for Grade 3 of the NICHD 2018 (6.47, 95% CI: 1.86–22.56) and Grade 3 of the NICHD 2019 (12.10, 95% CI: 2.52–58.05) (Figure 1B). Multivariate analysis for PHN at a PMA of 36 weeks based on each definition showed that PHN was associated with Grade 2 and Grade 3 BPD of the NICHD 2018 and NICHD 2019 criteria, with the highest adjusted OR observed for Grade 3 of the NICHD 2019 criteria (40.37, 95% CI: 5.15–316.35) (Figure 2).

## Discussion

In this study, the diagnostic criteria for BPD established in 2001, and newly proposed criteria established in 2018 and 2019 were used to define the severity of the disease considering the association of PHN at 36 weeks of PMA and later health outcomes of preterm infants born at < 32 weeks' gestation. A notable finding of the current study is that the association of PHN at a PMA of 36 weeks was well demonstrated in the newer criteria, especially in the NICHD 2019 criteria. To the best of our knowledge, this is the first study that compared different criteria of BPD regarding PHN in preterm infants.

This finding is important in terms of the pathophysiology of the disease as BPD is now recognized as a form of pulmonary vascular disease in premature infants. Abnormalities in the development of pulmonary blood vessels, along with lung damage, could lead to impairment in the structure and function of pulmonary blood vessels (16). As a result, pulmonary hypertension could develop in preterm infants as a spectrum of vascular disease, which is frequently accompanied by a severe form of BPD and is associated with poor outcomes (12). Therefore, the NICHD 2019 criteria might discriminate more severe diseases and reflect the pathophysiology of chronic lung disease in prematurity when compared with previous criteria.

Many previous studies have compared the prognosis of BPD based on various diagnostic criteria. Vyas-Read S. et al. showed findings from the Children's Hospitals Neonatal Consortium that the NICHD 2019 criteria had the highest odds of mortality or tracheostomy as short-term outcomes, followed by the NICHD 2018 criteria (17). A single-center retrospective study of infants born at a GA <32 weeks from China reported that the NICHD 2001 criteria had a lower specificity and worse positive predictive value than the NICHD 2018 criteria regarding severe respiratory morbidities or death at 18–24 months of CA (18).

A study using nationwide registered data of extremely preterm infants in Korea to compare three definitions of BPD showed that the NICHD 2018 or NICHD 2019 criteria had better prediction in both respiratory morbidities and neurodevelopmental outcomes at 18–24 months of CA (19). Another study using nationwide data of infants with <32 weeks' gestation also compared the NICHD 2001 and NICHD 2019 criteria and showed that the latter was associated more with adverse respiratory and neurodevelopmental outcomes at 2 years of age (20). A previous study that analyzed the association of different definitions of BPD with economic impact showed that the NICHD 2019 criteria had the strongest correlation with hospital charges and the 1st year of life-associated hospital charges for preterm infants (21). The results of the present study are compatible with those of previous studies in that the association of BPD with long-term outcomes was not evident in the NICHD 2001 criteria, while both the NICHD 2018 and NICHD 2019 criteria had associations with those outcomes (22, 23).

Given the nature of the criteria, better prediction of later outcomes in the NICHD 2019 criteria seems obvious, as they were originally determined based on the best predictive ability among various pre-specified definitions of BPD (10). Further, in NICHD 2019 definition, the best predictability was found when BPD was graded according to the mode of respiratory support regardless of oxygen use. These findings are intriguing, as a previous study reported that a higher capillary partial pressure of carbon dioxide, rather than the requirement for oxygen, was a good predictor of later respiratory outcomes among patients with BPD (24).

There are several limitations to this study. First, this was a single-center study; therefore, the number of participants was smaller than the corresponding of large-scale studies (10, 25). Second, only 74.3% of the study population was tested using Bayley-III at a CA of 18–24 months, although other neurodevelopmental outcomes, such as CP, hearing impairment, and blindness, have been well reported. Other aspects of respiratory outcomes, such as respiratory medication and respiratory symptoms, were not assessed. The incidence of PHN in patients with BPD was 9.2%, which was relatively lower than that in a previous study (11). This might be attributed to the study population, which was a relatively mature population of GA <32 weeks in this study. Furthermore, socioeconomic and environmental factors that influence respiratory and neurodevelopmental outcomes were not included in this study (11).

In conclusion, recently suggested BPD criteria, such as the NICHD 2018 and NICHD 2019 criteria, showed an association between the severity of BPD and later outcomes in preterm infants. Furthermore, the association of BPD severity based on these recent criteria and PHN was well demonstrated in this study. As PHN is an important aspect of the pathophysiology of BPD, it might be speculated that recent criteria of BPD could discriminate severity of disease not only more practically but also based on the entity of BPD.

## Data Availability

The raw data supporting the conclusions of this article will be made available by the authors, without undue reservation.
