# Supplementary material for: Association of newer definitions of bronchopulmonary dysplasia with pulmonary hypertension and long-term outcomes
Source: Front Pediatr. 2023 Feb 15;11:1108925. doi: 10.3389/fped.2023.1108925 (PMC9977292; doi:10.3389/fped.2023.1108925)
Supplement: Supplementary file 1 [file Table1.docx]

Supplementary materials

**Association of newer definitions of bronchopulmonary dysplasia with pulmonary hypertension and long-term outcomes**

Jae Kyoon Hwang MD^a^, Seung Han Shin MD, PhD^b^, Ee-Kyung Kim MD, PhD^b^, Seh Hyun Kim MD^b,^ Han-Suk Kim MD, PhD^b^.

^a^ Department of Pediatrics, Hanyang University Guri Hospital, Gyeonggi-do, Republic of Korea

^b^ Department of Pediatrics, Seoul National University College of Medicine, Seoul, Republic of Korea

Corresponding Author: Seung Han Shin, MD.

Mailing address: Department of Pediatrics, Seoul National University Children's Hospital, 101 Daehak-ro Jongno-gu, Seoul 03080, Republic of Korea

Telephone number: +82-2-2072-3555, Fax number: +82-2-2072-0590

Email: [revival421@snu.ac.kr](mailto:revival421@snu.ac.kr)

Supplementary Table S1. Definition of BPD of the NICHD (2001, 2018, 2019) criteria based on the mode of respiratory support and FiO_2_ ranges

|  | Invasive IPPV | N-CPAP  or NIPPV | Nasal cannula | | | |
| --- | --- | --- | --- | --- | --- | --- |
|  |  |  | ≥ 3 L/min | 2–3 L/min | 1–2 L/min | < 1 L/min |
| 2019  NICHD | III | II | | | I | |
| 2001 Mild | - | | 21 | | | |
| 2001 Moderate | - | | 22–29 | | | |
| 2001 Severe | ALL | | ≥ 30 | | | |
| 2018 I | - | 21 | | 22–29 | | 22–70 |
| 2018 II | 21 | 22–29 | | ≥ 30 | | > 70 |
| 2018 III | > 21 | ≥ 30 | | - | | |

BPD, bronchopulmonary dysplasia; NICHD, National Institute of Child Health and Human Development; IPPV, intermittent positive pressure ventilation; N-CPAP, nasal continuous positive airway pressure; NIPPV, noninvasive positive pressure ventilation

Cf) NICHD 2018 Grade IIIA ; Early death (14 days and 36 weeks) owing to lung disease that cannot be attributable to other neonatal morbidities

Supplementary Table S2. Demographics of the study population according to the severity of bronchopulmonary dysplasia (NICHD 2001)

|  | No BPD (n=202) | Mild BPD (n=67) | Moderate BPD (n=32) | Severe BPD (n=53) | p-value |
| --- | --- | --- | --- | --- | --- |
| GA (week) | 30.7 (30–31.3) | ^§^27.6 (26.6–28.6) | ^§^28.2 (26.4–29.5) | ^§^26.9 (25.3–29) | <0.001 |
| Birth weight (g) | ^§^1380 (1230–1590) | ^§^1000 (850–1170) | ^§^990 (790–1220) | ^§,*^730 (620–980) | <0.001 |
| Birth weight z-score | -0.2 (-0.6–0.3) | 0.1 (-0.3–0.5) | -0.1 (-1–0.5) | -0.3 (-1.1–0.3) | 0.021 |
| SGA | 16 (7.9) | 3 (4.5) | 6 (18.8) | 11 (20.8) | 0.006 |
| Male | 101 (50) | 35 (52.2) | 20 (62.5) | 23 (43.4) | 0.394 |
| C/S | 114 (56.4) | 41 (61.2) | 16 (50) | 35 (66) | 0.443 |
| Multiple birth | 150 (74.3) | 40 (59.7) | 13 (40.6) | 24 (45.3) | <0.001 |
| hCAM | 67 (33.3) | 36 (54.6) | 14 (45.2) | 33 (62.3) | <0.001 |
| PROM | 78 (38.8) | 31 (47) | 13 (40.6) | 25 (48.1) | 0.499 |
| Oligohydramnios | 32 (15.8) | 16 (23.9) | 8 (25) | 19 (35.9) | 0.013 |
| Antenatal steroid | 170 (84.2) | 57 (85.1) | 30 (93.8) | 50 (94.3) | 0.150 |

Values are expressed as number (%) or median (interquartile range). BPD, bronchopulmonary dysplasia; NICHD, National Institute of Child Health and Human Development; GA, gestational age; SGA, small for gestational age; C/S, Cesarean section; hCAM, histologic chorioamnionitis; PROM, premature rupture of membranes. ^§^ indicates p<0.013 compared with the no BPD group in the post-hoc analysis with Bonferroni correction. ^*^ indicates p<0.013 compared with the mild BPD group in the post-hoc analysis with Bonferroni correction.

Supplementary Table S3. Demographics of the study population according to the severity of bronchopulmonary dysplasia (NICHD 2018)

|  | No BPD (n=269) | Grade 1 (n=47) | Grade 2 (n=15) | Grade 3 (n=23) | p-value |
| --- | --- | --- | --- | --- | --- |
| GA (week) | 30.3 (28.9–31.1) | 28.3 (26.6–29.7) | 26.0 (24.3–27.7) | 26.4 (24.9–28.7) | <0.001 |
| Birth weight (g) | 1290 (1075–1500) | 990 (780–1230) | 630 (590–890) | 710 (620–800) | <0.001 |
| Birth weight z-score | -0.1 (-0.6–0.4) | -0.1 (-1.0–0.3) | -0.4 (-1.8–0.8) | -0.3 (-1.1–0.2) | 0.040 |
| SGA | 19 (7.1) | 8 (17.0) | 5 (33.3) | 4 (17.4) | 0.001 |
| Male | 136 (50.6) | 26 (55.3) | 10 (66.7) | 7 (30.4) | 0.127 |
| C/S | 155 (57.6) | 28 (59.6) | 9 (60.0) | 14 (60.9) | 0.984 |
| Multiple birth | 190 (70.6) | 21 (44.7) | 8 (53.3) | 8 (34.8) | <0.001 |
| hCAM | 103 (38.6) | 20 (43.5) | 10 (66.7) | 17 (73.9) | 0.002 |
| PROM | 109 (40.8) | 18 (38.3) | 7 (50.0) | 13 (56.5) | 0.430 |
| Oligohydramnios | 48 (17.8) | 8 (17.0) | 6 (40.0) | 13 (56.5) | <0.001 |
| Antenatal steroid | 227 (84.4) | 44 (93.6) | 15 (100.0) | 21 (91.3) | 0.115 |

Values are expressed as number (%) or median (interquartile range). BPD, bronchopulmonary dysplasia; NICHD, National Institute of Child Health and Human Development; GA, gestational age; SGA, small for gestational age; C/S, Cesarean section; hCAM, histologic chorioamnionitis; PROM, premature rupture of membranes

Supplementary Table S4. Clinical courses according to the severity of BPD (NICHD 2001)

|  | No BPD (n=202) | Mild BPD (n=67) | Moderate BPD (n=32) | Severe BPD (n=53) | p-value |
| --- | --- | --- | --- | --- | --- |
| RDS | 67 (33.2) | 51 (76.1) | 24 (75) | 43 (81.1) | <0.001 |
| PDA treated | 24 (17.8) | 32 (48.5) | 17 (54.8) | 35 (68.6) | <0.001 |
| IVH (grade ≥3) | 2 (1) | 2 (3) | 1 (3.1) | 6 (11.3) | 0.004 |
| NEC | 3 (1.5) | 4 (6) | 1 (3.1) | 6 (11.3) | 0.007 |
| ROP operation | 2 (1) | 3 (4.5) | 4 (12.5) | 23 (43.4) | <0.001 |
| PHN at PMA 36 weeks | 1 (1.4) | 1 (1.5) | 3 (9.4) | 10 (18.9) | <0.001 |
| Re-hospitalization | 27 (13.4) | 8 (11.9) | 11 (34.4) | 21 (39.6) | <0.001 |
| Combined NDI | 16 (7.9) | 8 (11.9) | 5 (15.6) | 21 (39.6) | <0.001 |

Values are expressed as number (%) or median (interquartile range). BPD, bronchopulmonary dysplasia; NICHD, National Institute of Child Health and Human Development; RDS, respiratory distress syndrome; PDA, patent ductus arteriosus; IVH, intraventricular hemorrhage; NEC, necrotizing enterocolitis; ROP, retinopathy of prematurity; PHN, pulmonary hypertension; PMA, postmenstrual age; NDI; neurodevelopmental impairment

Supplementary Table S5. Clinical courses according to the severity of BPD (NICHD 2018)

|  | No BPD (n=269) | Grade 1 (n=47) | Grade 2 (n=15) | Grade 3 (n=23) | p-value |
| --- | --- | --- | --- | --- | --- |
| RDS | 118 (43.9) | 35 (74.5) | 14 (93.3) | 18 (78.3) | <0.001 |
| PDA treated | 56 (27.9) | 27 (61.4) | 9 (60) | 16 (69.6) | <0.001 |
| IVH (grade ≥3) | 4 (1.5) | 3 (6.4) | 1 (6.7) | 3 (13) | 0.006 |
| NEC | 7 (2.6) | 3 (6.4) | 2 (13.3) | 2 (8.7) | 0.077 |
| ROP operation | 5 (1.9) | 8 (17) | 8 (53.3) | 11 (47.8) | <0.001 |
| PHN at PMA 36 weeks | 2 (1.4) | 4 (8.5) | 3 (20) | 6 (26.1) | <0.001 |
| Re-hospitalization | 35 (13.0) | 14 (29.8) | 5 (33.3) | 13 (56.5) | <0.001 |
| Combined NDI | 24 (8.9) | 9 (19.1) | 6 (40.0) | 11 (47.8) | <0.001 |

Values are expressed as number (%) or median (interquartile range). BPD, bronchopulmonary dysplasia; NICHD, National Institute of Child Health and Human Development; RDS, respiratory distress syndrome; PDA, patent ductus arteriosus; IVH, intraventricular hemorrhage; NEC, necrotizing enterocolitis; ROP, retinopathy of prematurity; PHN, pulmonary hypertension; PMA, postmenstrual age; NDI; neurodevelopmental impairment

**Figure legend**

Figure S1. Flow chart of study population. GA, gestational age; FU, follow-up; CA, corrected age
