# Supplementary figures and images for: Association of newer definitions of bronchopulmonary dysplasia with pulmonary hypertension and long-term outcomes
Source: Front Pediatr. 2023 Feb 15;11:1108925. doi: 10.3389/fped.2023.1108925 (PMC9977292; doi:10.3389/fped.2023.1108925)

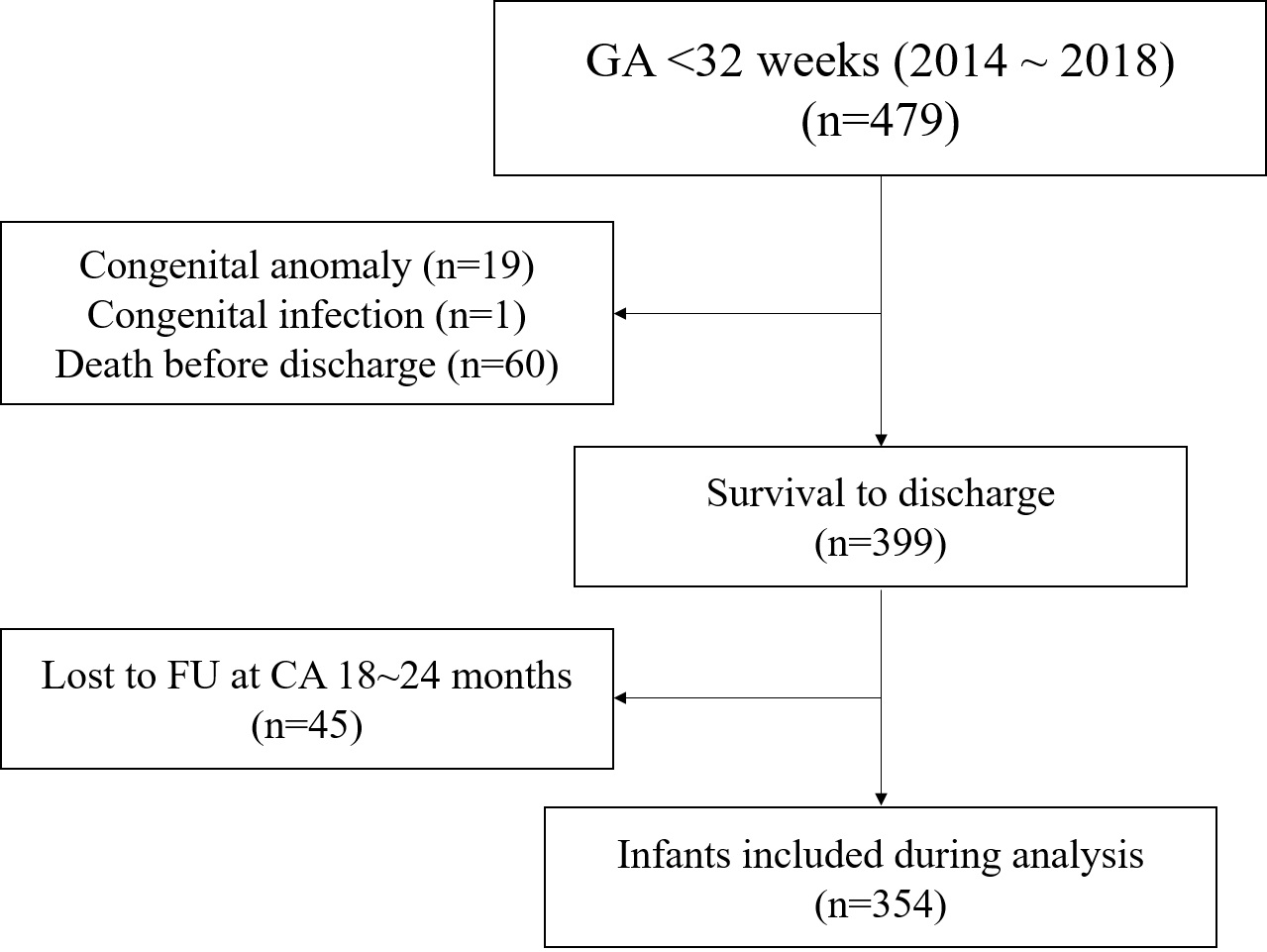

Supplement: Supplementary file 2 [file Image1.tif]
